# A Novel Pyramid Network with Feature Fusion and Disentanglement for Object Detection

**DOI:** 10.1155/2021/6685954

**Published:** 2021-03-15

**Authors:** Guoyi Yu, You Wu, Jing Xiao, Yang Cao

**Affiliations:** ^1^School of Computer Science, South China Normal University, Guangzhou 510631, China; ^2^School of Mathematics and Statistics, Hunan Normal University, Changsha 410081, China

## Abstract

In order to alleviate the scale variation problem in object detection, many feature pyramid networks are developed. In this paper, we rethink the issues existing in current methods and design a more effective module for feature fusion, called multiflow feature fusion module (MF^3^M). We first construct gate modules and multiple information flows in MF^3^M to avoid information redundancy and enhance the completeness and accuracy of information transfer between feature maps. Furtherore, in order to reduce the discrepancy of classification and regression in object detection, a modified deformable convolution which is termed task adaptive convolution (TaConv) is proposed in this study. Different offsets and masks are predicted to achieve the disentanglement of features for classification and regression in TaConv. By integrating the above two designs, we build a novel feature pyramid network with feature fusion and disentanglement (FFAD) which can mitigate the scale misalignment and task misalignment simultaneously. Experimental results show that FFAD can boost the performance in most models.

## 1. Introduction

Object detection is one of the most important and challenging tasks in the field of computer vision. This task widely benefits image/video retrieval, intelligent surveillance, and autonomous driving. Although the performance of object detector grows rapidly with the development of deep convolutional neural networks, the existing detectors still suffer from the problems caused by the scale variation across object instances. To resolve this issue, the image pyramid method [1] takes pictures of different resolutions as input to improve the robustness of the model to small objects. However, this strategy greatly increases the amount of memory and computation. In SSD [2], the authors propose a method to detect objects of different sizes on feature maps at different levels. Compared with the solution that uses an image pyramid, this method has less memory and computational cost. Unfortunately, the performance of small object detection is still poor, since the features in low layers of the convolutional network always contain more geometric information and less semantic information. To alleviate this problem, FPN [3] creates a top-down architecture with lateral connections for building high-level semantic feature maps at all scales. Recently, the assistance of geometric information in shallow layers to large object detection is noticed. Several methods such as PANet [4] and BiFPN [5] add an extra bottom-up information flow path based on FPN to enhance the deep-layer features with accurate localization signals existing in low levels. Several methods like Libra RCNN [6] and M2det [7] first gather multilayer features into one layer and finally split it into a feature pyramid to integrate geometric and semantic information.

**Figure 1:**
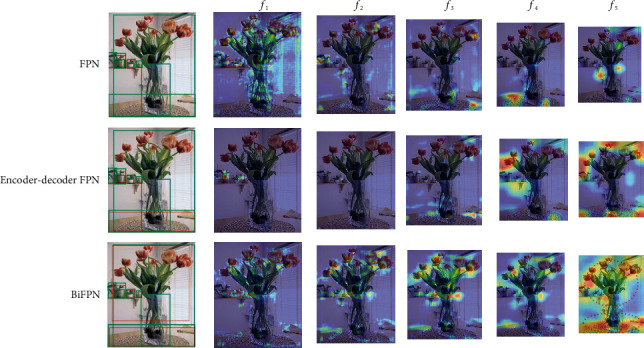
Heatmap visualization examples of current fusion methods. *f*_*i*_, *i* = 1...5, means the output feature of *i*-th level in pyramid network. Green boxes: ground truth; red boxes: detection result.

Despite the performance gained by the above pyramidal architecture, they still have some intrinsic limitations. Most feature pyramid networks are constructed by simply aggregating the features of different levels intuitively, which ignore the intrinsic properties between the features of different levels. SPEC [8] shows us that the similarity between adjacent feature maps is high, while those far apart are opposite. In this paper, we observe that there are two critical drawbacks existing in most previous fusion methods. First, information redundancy problem caused by directly summing or concatenating feature maps hinders the performance of detection. Second, it is difficult to accurately transfer information between feature maps, especially for feature maps that are far apart, which leads to the loss of some targets.  Figure 1 demonstrates the heatmap visualization examples of multilevel features after various feature pyramid networks. We can observe the following: (1) Only a few features are captured by conventional FPN and it has no response to large-scale objects. (2) The second method has larger activation regions at deep layers, but it contains some inaccurate information. (3) Although the third method has better performance on both large and small objects, it still misses several targets and has some unnecessary noise. Further, ignoring the spatial misalignment between classification and localization functions, the output of most pyramidal networks is shared by downstream head of detector. Some researches [9–11] have revealed that the spatial sensitivities of classification and localization on the feature maps are different, which can limit the performance of detection. However, previous solutions to this problem can be deemed to disentangle the information by adding a new branch and essentially increase the parameters of the head. The conflict between the two tasks is still not eliminated, since the feature map extracted by backbone is still shared by the two branches, which motivates us to explore a feature pyramid architecture with spatial disentanglement.

**Figure 2:**
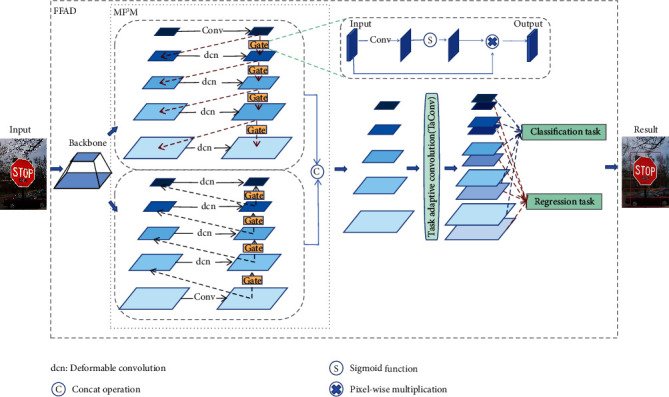
The overview of the proposed FFAD cooperated with single-stage detector. The features of input images are first extracted by the backbone network, and then MF^3^M fuse these features through multiple paths. Finally, TaConv produces a multilevel feature pyramid. There are two parallel feature maps used to predict specific categories and regress precise boxes, respectively, at each level.

In this paper, we aim to propose a novel feature pyramid network to break the above bottleneck restrictions. As shown in Figure 2, we firstly construct two subnetworks for top-down information flow and down-top information flow. Then, following the attention mechanism applied in these works [12–15] and the feature selection method on high-dimensional data [16], we set several gate modules to help the network focusing on important features as well as suppressing unnecessary ones. Moreover, we add an extra fusion path in each direction for enhancing the power of communication to prevent the loss of important information. Finally, we gather up the fusion outputs of two subnetworks. It is worth noting that there are five information flow paths in our module: one is horizontal, and the others are vertical. In order to alleviate the inherent conflict between classification and regression in feature pyramid, a modified deformable convolution is proposed for feature decoupling, called task-adaptive convolution (TaConv). By predicting two sets of offsets and mask, respectively, TaConv outputs two feature maps for classification and regression, respectively, at each level of feature pyramid. Our method brings significant performance improvement compared with the state-of-the-art one-stage object detectors.

The contributions of this study are as follows:

1. We rethink the limitation existing in previous feature fusion strategies and design a more effective module to avoid these issues.
2. We further propose a method (TaConv) for the feature decoupling in one-stage detector to alleviate the discrepancy between classification and regression.
3. We construct a novel feature pyramid network with feature fusion and decoupling and validate the effectiveness of our approach on the standard MS-COCO benchmark. The proposed network can boost the performance of most single-shot detectors (by about 1∼2.5AP).

## 2. Related Work

### 2.1. Object Detection

There are mainly two streams of methods in object detection. The first stream is two-stage. Methods in this stream include RCNN family [17–19]. R-FCN [20] and Mask RCNN [21] consist of a separate region proposal network and a region-wise prediction network. They firstly predict region proposals and then classify and fine-tune each of them. Methods in the other stream are one-stage. This type of detector directly predicts objects category and coordinates at each pixel of feature map; thus, the efficiency of such methods is higher than that of two-stage ones. However, one-stage detectors in early time such as SSD [2] and YOLO family [22–24] lagged behind two-stage detectors as regards the performance. With the advent of focal loss [25], the category imbalance problem in the single-stage detector is greatly alleviated. Since then, following works [26–28] further improve its performance by designing more elaborate heads. At present, the single-stage detectors can achieve performance that is very close to that of the two-stage ones.

### 2.2. Feature Fusion

Due to the convolutional networks' deepening and downsampling operations, the features of small objects are always lost. To tackle this problem, two strategies were proposed in the literature. The first one is image pyramid method such as SNIP [1] and SNIPER [29]. These methods take pictures of different resolutions as input and perform detection separately and combine these prediction results to give the final results. The other strategy is feature pyramid. These methods like SSD [2] and MS-CNN [30] conduct small object detection directly on the shallow feature maps and perform large object detection on the deep feature maps. Compared with the first strategy, the additional memory and computational cost required by the second strategy are greatly reduced, so it can be deployed during the training and testing phase of the real-time network. Moreover, low-level features generally lack semantic information but are rich in keeping geometric details while high-level features are opposite. Therefore, an effective feature fusion strategy plays a crucial role in processing features of objects with various scales. FPN [3], the milestone of pyramidal network, propagates high-level semantic information to shallow level by building a top-down architecture. Since then, feature pyramid has been widely used in the object detection task. Recently, considering the lack of geometric information of deep layer features, several bidirectional models such as PANet [4] and BiFPN [5] add a down-top path for low-level feature maps aggregation based on the FPN. Libra-RCNN [6] firstly fuses features of all layers and then disentangles them into the pyramid. M2Det [7] stacks several U-shaped modules to fuse multilayer features followed by generating the feature pyramid. Moreover, different from the above method, there are some other approaches that fuse features by concatenating features from different layers in the forward propagation of the backbone. For instance, Hourglass Network [31] concatenates features with the previous layers in the repeated bottom-up and top-down processes. HRNet [32] gradually adds a low-resolution subnetwork to the high-resolution major network in parallel.

### 2.3. Feature Disentanglement

Most object detectors share the features extracted by the backbone for both classification and bounding box regression; thus, there is a lack of understanding between the two tasks. There has been some work on the conflict between the classification and regression tasks. Zhang and Wang [33] point out that the direction of the two task gradients is inconsistent, implying the potential conflicts between the two tasks gradients. IoU-Net [9] alleviates this discrepancy by adding an extra head to predict the localization confidence and then aggregates it with the classification confidence together to be the final score. Double-Head RCNN [10] disentangles the sibling head into two specific branches for classification and localization. TSD [11] shows that classification task pays more attention to the features in the salient areas of objects, while the features around the boundary are beneficial for bounding box regression. The authors ease this issue by generating two disentangled proposals for classification and localization, respectively. Despite the fact that the satisfactory performance can be obtained by this detection head disentanglement, the conflict between the two tasks still remains, since the inputs to the two heads are still shared. In this paper, we propose a novel feature pyramid network with feature fusion and disentanglement called FFAD, which can alleviate the scale misalignment and task misalignment simultaneously. To the best of our knowledge, there is currently no work to explore spatial decoupling of feature pyramids.

## 3. Proposed Method

FFAD contains two submodules, that is, MF^3^M and TaConv. Compared with most of the current methods, MF^3^M aggregates features more effectively. Then the output feature maps of MF^3^M are disentangled by TaConv for alleviating inherent conflict between the classification and regression task. The prediction of classical pyramidal networks can be written as

(1) $$\begin{array}{c} P_{c}=H_{c}\left(F_{i}\right),\;i=1...L,\\ P_{r}=H_{r}\left(F_{i}\right),\;i=1...L, \end{array}$$

where *P*_*c*_ and *P*_*r*_ denote the classification results and regression results, respectively; *H*_*c*_ and *H*_*r*_ are the heads for transforming feature to specific category and localization of object; *F*_*i*_ denotes the feature map of *i-*th level in feature pyramid, and *L* denotes the numbers of layers of feature pyramid. Unlike conventional pyramidal networks, FFAD produces two feature maps for two tasks, respectively, at each level of the feature pyramid:

(2) $$\begin{array}{c} P_{c}=H_{c}\left(F_{i}^{c}\right),\;i=1\ldots L,\\ P_{r}=H_{r}\left(F_{i}^{r}\right),\;i=1\ldots L, \end{array}$$

where *F*_*i*_^*c*^ and *F*_*i*_^*r*^ denote the feature map for classification and regression of the *i*-th layer in FFAD, respectively.

### 3.1. Multiflow Feature Fusion Module

**Figure 3:**
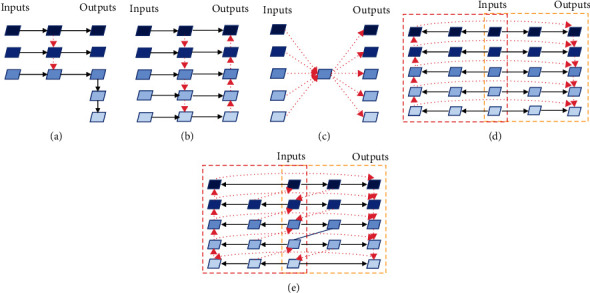
We propose two general structures of multiflow feature fusion methods: (d) 3-flow structure and (e) 5-flow structure. Native FPN (a), bidirectional FPN (b), and encoder-decoder FPN (c) are some other popular fusion methods. Red-dotted lines mean that they can be several operations including upsampling, downsampling, summing, and concatenation. The different directions of the red-dotted lines represent different information flows. Each solid black line presents an independent convolution. The red-dotted box represents the down-top subnetwork and the yellow-dotted box represents the top-down subnetwork.

We conclude that there are about three styles of feature pyramid networks: (1) conventional FPNs that are single directional pyramid network (as shown in Figure 3(a)), (2) bidirectional pyramid networks (as shown in Figure 3(b)), and (3) encoder-decoder FPNs (as shown in Figure 3(c)). As shown in Figure 3(d), the parts in the red- and yellow-dotted boxes represent two subnetworks in different directions that share inputs. There are three feature nodes at each level of each subnetwork. Further, we propose information augmentation for enhancing the signal transmitted between feature nodes, especially those that are far apart. As seen from Figure 3(e), in the top-down subnetwork, both the second and third nodes of each layer have a fusion with the shallower features except for the shallowest. Meanwhile, in the down-top subnetwork, the second and third nodes of each layer are fused with the deeper features except for the deepest layer. At the same time, in order to simplify the network, we remove the shallowest second node in the top-down network and the deepest second node in the down-top network, so that there is only one input edge. It is worth noting that there are two information flow paths in each subnetwork. Finally, we gather up the outputs of two subnetworks to form the fifth information flow. Let *x*_*i*_ be the *i*-th input of MF^3^M and let *y*_*i*_ be the *i-*th output of MF^3^M. Then the output of the MF^3^M is

(3) $$\begin{array}{c} y_{i}=conv\left(C\left(F_{t-d}\left(x_{i}\right),F_{d-t}\left(x_{i}\right)\right)\right), \end{array}$$

where *conv*(*·*) denotes the convolution operation, *C*(*·*) denotes the concatenation operation, and *F*_*t*−*d*_(*·*) and *F*_*d*−*t*_(*·*) are the outputs of top-down and down-top subnetworks, respectively:

(4) $$\begin{array}{c} F_{t-d}\left(x_{i}\right)=conv\left(conv\left(x_{i}\right)+M\left(conv\left(x_{i-1}\right)\right)\right)+M\left(F_{t-d}\left(x_{i-1}\right)\right),\\ F_{d-t}\left(x_{i}\right)=conv\left(conv\left(x_{i}\right)+U\left(conv\left(x_{i-1}\right)\right)\right)+U\left(F_{d-t}\left(x_{i-1}\right)\right), \end{array}$$

where *M*(*·*) is the max-pooling layer and *U*(*·*) is the bilinear upsampling layer.

EfficientDet [5] already shows that the feature map of different scales should have a different contribution to the output and proposes adding a weight for each input feature, while most previous methods treat all input features equally without distinction. Inspired by the spatial attention mechanism and the intrinsic connections between feature maps, we design a simple gate module for controlling the intensity of information flow. Thus, the outputs of top-down and down-top subnetworks are as follows:

(5) $$\begin{array}{c} F_{t-d}\left(x_{i}\right)=g\left(conv\left(conv\left(x_{i}\right)+M\left(conv\left(x_{i-1}\right)\right)\right)\right)+g\left(M\left(F_{t-d}\left(x_{i-1}\right)\right)\right),\\ F_{d-t}\left(x_{i}\right)=g\left(conv\left(conv\left(x_{i}\right)+U\left(conv\left(x_{i-1}\right)\right)\right)\right)+g\left(U\left(F_{d-t}\left(x_{i-1}\right)\right)\right), \end{array}$$

where *g*(*·*) can be written as

(6) $$\begin{array}{c} g\left(x\right)=sigmoid\left(conv\left(x\right)\right)⊗x, \end{array}$$

and *x* represents the input; ⊗ denotes pixel-wise multiplication.

Deformable convolution is often embedded in the backbone as well as the last layer of detector towers to further improve the performance of object detectors. In order to further improve the feature pyramid network, we use DCN [34] to adjust the results after fusing with other layer features in the pyramid network. To avoid the extra computing cost caused by deformable convolution as far as possible, we only embed it in the nodes of each layer after the first fusion with other layers. In this way, the outputs of top-down and down-top subnetworks, *F*_*t*−*d*_(*·*) and *F*_*d*−*t*_(*·*), can be formulated as follows:

(7) $$\begin{array}{c} F_{t-d}\left(x_{i}\right)=\left\{\begin{array}{cc} g\left(conv\left(conv\left(x_{i}\right)\right)\right), & i=1,\\ g\left(dc\;onv\left(conv\left(x_{i}\right)+M\left(conv\left(x_{i-1}\right)\right)\right)\right)+g\left(M\left(F_{t-d}\left(x_{i-1}\right)\right)\right), & i=2\ldots 5, \end{array}\right)\\ F_{d-t}\left(x_{i}\right)=\left\{\begin{array}{cc} g\left(dc\;onv\left(conv\left(x_{i}\right)+U\left(conv\left(x_{i-1}\right)\right)\right)\right)+g\left(U\left(F_{d-t}\left(x_{i-1}\right)\right)\right), & i=1\ldots 4,\\ g\left(conv\left(conv\left(x_{i}\right)\right)\right), & i=5, \end{array}\right) \end{array}$$

where *dc*  *onv*(*·*) denotes deformable convolution operation.

### 3.2. Task-Adaptive Convolution

**Figure 4:**
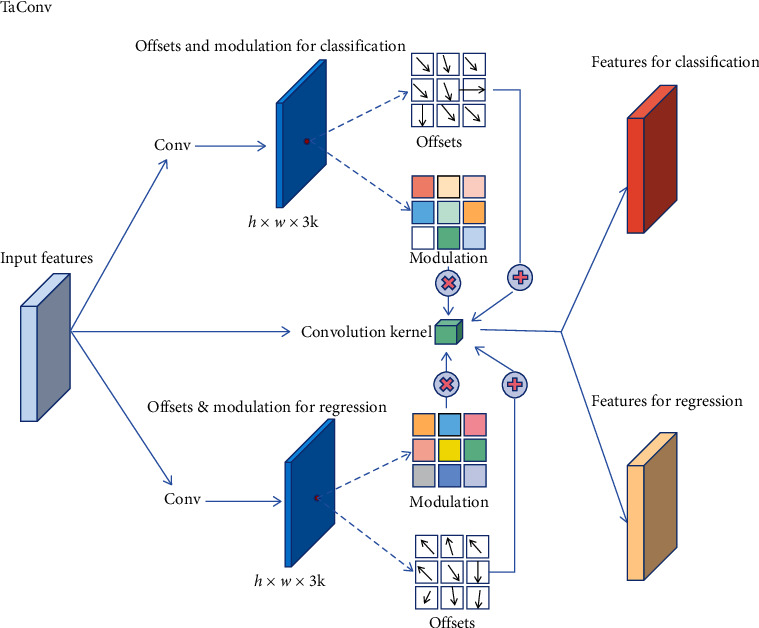
Structural details of task-adaptive convolution (TaConv).

To mitigate the misalignment between classification and localization existing in classical feature pyramids, we propose task-adaptive convolution. It is indeed a modified modulated deformable convolution. We borrow the idea of DCN [34] to distinguish between features suitable for classification and suitable for regression, due to its superior ability to capture the key information of objects. As shown in Figure 4, for the features of each level in feature pyramids, TaConv first predicts two groups of offsets and modulations. Then the two groups of offsets are added to the coordinates of each sampling point of the convolution kernel, respectively. The two modulations are multiplied by the value of each sampling point of the convolution kernel. Finally, TaConv generates two independent feature maps: one is sensitive for classification task, and the other is sensitive to localization task. Let *x* represent the pixel value of feature map and the outputs of TaConv can be formulated as follows:

(8) $$\begin{array}{c} F_{i}^{c}=\sum_{k=1}^{K}w_{k}\cdot x_{i}\left(p_{x}+\Delta p_{x}^{c},p_{y}+\Delta p_{y}^{c}\right)\cdot m_{c},\\ F_{i}^{c}=\sum_{k=1}^{K}w_{k}\cdot x_{i}\left(p_{x}+\Delta p_{x}^{r},p_{y}+\Delta p_{y}^{r}\right)\cdot m_{r}, \end{array}$$

where *K* denotes the size of convolution kernel; *w*_*k*_ denotes the *k-*th point of kernel. *p*_*x*_ and *p*_*y*_ denote the horizontal and vertical coordinates of sampling point. Δ*p*_*x*_^*c*^ and Δ*p*_*y*_^*c*^ represent the deviation of the classification task on the *X*-axis and *Y*-axis, respectively. Δ*p*_*x*_^*r*^ and Δ*p*_*y*_^*r*^ denote the deviation of the regression task on the *X*-axis and *Y*-axis, respectively. *m*_*c*_ and *m*_*r*_ are the modulation multiplied by the convolution kernel parameters.

## 4. Experimental Evaluation

We perform our experiments on the challenging MS-COCO [35] benchmark of 80-category. Following the standard protocol [36], we train on the training set (consisting of around 118k images) and then report the results of minival set (consisting of 5k images) for ablation studies. To compare the accuracy of our algorithm with those of the state-of-the-art single-shot detectors, we also report results of test-dev set (consisting of around 2k images) which has no public labels and requires the use of the evaluation server.

### 4.1. Implementation Details

In our study, we embed our method into several latest and state-of-the-art single-stage detectors including RetinaNet [25], FCOS [26], and ATSS [28]. For fair comparison with the above detectors, the configuration of hyperparameters used in our experiments is set as same as the literature's. Specifically, we use the ImageNet [37] pretrained models such as ResNet-50 [38] followed by FPN structure as the backbone. We use the Stochastic Gradient Descent (SGD) algorithm to optimize the training loss for 180k iterations with 0.9 momentum, 0.0001 weight decay, and a mini-batch of 8 images. The initial learning rate is set to 0.05 and we reduce the learning rate by a factor of 10 at iterations of 120k and 160k, respectively. Unless otherwise stated, the input images are resized to have their shorter side being 800 and their longer side less or equal to 1333. We do not use any noise reduction method, and no data augmentations except standard horizontal flipping are used. During the inference stage, we resize the input image in the same way as in the training stage and postprocess the predicted bounding boxes with a predicted class obtained by forwarding images through the network, using the same hyperparameters of the above detectors.

### 4.2. Ablation Study

**Figure 5:**
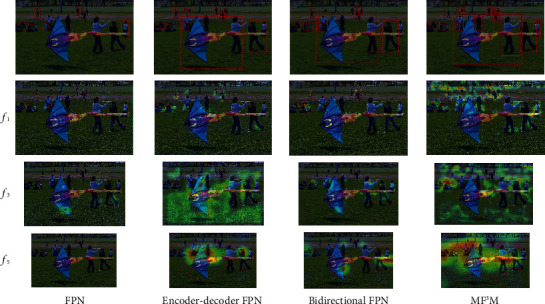
Heatmap visualization examples of our proposed method MF^3^M and other current fusion methods embedded in RetinaNet. *f*_*i*_, *i* = 1, 3, 5, means the output feature of *i*-th level in pyramid network.

To demonstrate that our proposed MF^3^M can capture the objects' features of different sizes more effectively, we compare MF^3^M with other common feature fusion modules on FCOS. The results are shown in Table 1. Compared with the baseline that actually uses single directional FPN (37.1 AP), encoder-decoder FPN obtains a higher score (37.3 AP), especially with an increase of 0.4 AP for medium targets. Meanwhile, bidirectional FPN gives the best performance among these three common FPN styles (37.6 AP), and its large target detection is improved by 0.6 AP. Cooperating with 3 information flows' structure, detailed in Figure 3(d), the detector based on FCOS is promoted to 37.8 AP. This result verifies that splitting the series bidirectional structure into two unidirectional subnetworks can get better performance. By adding an additional information flow in each subnetwork, the performance of the detector is further improved by 0.5 AP. After fine-tuning the feature by DConv, our MF^3^M achieves 39.2 AP, outperforming most current feature fusion methods by a large margin. Specifically, the accuracy of detecting small objects (increased by 2.0 AP compared to the baseline) and large objects (increased by 3.4 AP compared to the baseline) is particularly improved. It is shown that our method can effectively fuse the features of cross-scale objects. In order to more intuitively observe the feature fusion ability of this method, we visualize the activation values of the features of FPN, bidirectional FPN, and MF^3^M. As shown in Figure 5, the first method loses some features of small objects and cannot detect large objects at all. Although the second and third methods can capture the feature of large objects and make progress in the detection of small objects, several objects are still missed. At the same time, our approach almost never misses features of both large and small targets.

**Figure 6:**
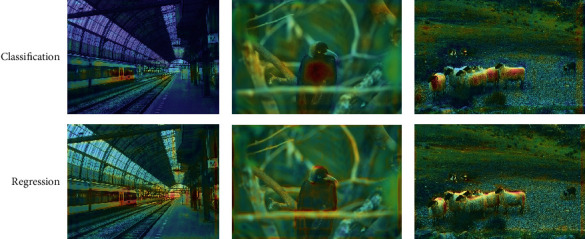
Visualization of the learned features from task-adaptive convolution. The first row indicates the features that are sensitive to classification. The second row indicates the features that are sensitive to regression.

As explained above, the core part of FFAD is composed of MF^3^M and task-adaptive convolution. The MF^3^M is responsible for computing feature maps, which contain rich features and the task-adaptive convolution decouples the features to make them task-sensitive.  Table 2 reports the detailed ablations on them to demonstrate their effectiveness. From the experimental results, we can know that this method can alleviate the conflict between the classification task and regression task to a certain extent. To better interpret what task-adaptive convolution learns, we visualize the learned feature on examples. As shown in Figure 6, the features of classification branch are more distributed in the central area of the objects, while the features of regression branch are more sensitive to the edge area of the objects.

### 4.3. Analysis of the Performance in Different DCN's Positions

**Figure 7:**
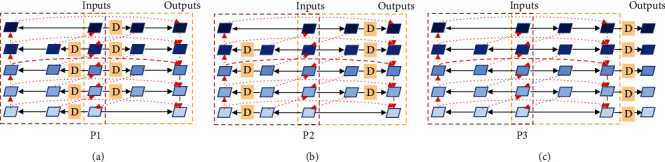
Different positions of deformable convolution in MF^3^F. The capital D stands for deformable convolution.

We have exhibited the effectiveness of MF^3^M for feature fusion and the deformable convolution plays a significant role in the adjustment of features. In this section, we further discuss the performance of MF^3^M with different deformable convolution's positions.  Figure 7 shows the structures where the deformable convolution is placed after the first to the third nodes of each layer in each subnet, respectively.  Table 3 illustrates that the scheme of P2, which uses DCN to fine-tune the nodes after the first feature fusion, has the best effect. We believe that better results can be achieved by fine-tuning all nodes after feature fusion with DCN. However, excessive use of DCN will bring greater computational effort, so we choose the most cost-effective scheme.

### 4.4. Compatibility with Other Single-Stage Detectors

Since FFAD has demonstrated its outstanding performance on FCOS with ResNet-50, we also present that it can still be effective when it is applied to other single-stage detectors. We directly conduct several experiments with different detectors including RetinaNet, FCOS, and ATSS on MS-COCO minival. All evaluation was performed on one Nvidia 1080Ti GPU. We set batch size to 8 and used the means of last 300 iterations in computation of speed. The results between the proposed FFAD and their original baselines are compared in Table 4. According to the first two columns of the table, it is obvious that FFAD can steadily improve the performance by 1.8∼2.6 AP, while the testing time is only increased by 3%∼11%.

### 4.5. Comparison with Other Feature Pyramids

With regard to various feature pyramidal models, we compare our FFAD with other state-of-the-art feature pyramid structures on FCOS.  Table 5 reports our experimental results. It is obvious that FFAD provides a dramatic performance increase compared to other advanced feature pyramid models, including PANet [4], HRNet [32], Libra [6], and NAS-FPN [39]. Moreover, FFAD also earns the close-to-the-minimum FLOPs increment among the feature pyramidal models.

### 4.6. Comparison with Other State-of-the-Art Detectors

In this section, we evaluate our proposed method on MS-COCO test-dev set and compare it with other state-of-the-art methods. For convenience, we only report FCOS equipped with our proposed FFAD. As shown in Table 6, it is observed that FFAD boosts the original baselines by a significant margin and achieves the state-of-the-art 49.5 AP using ResNext-101 backbone.

### 4.7. Visual Results

**Figure 8:**
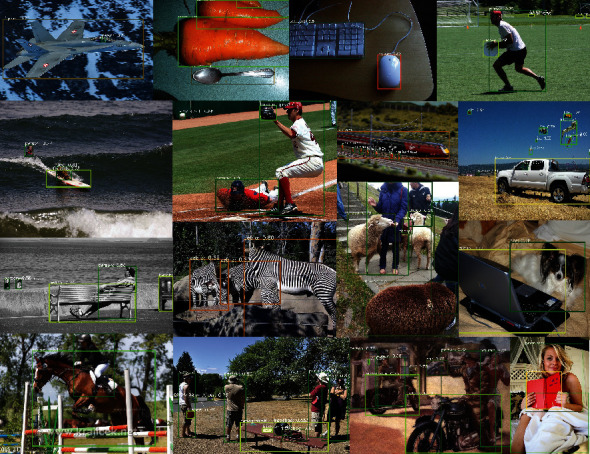
Visual results of FFAD on COCO minival split.

We visualize part of the detection results of our FFAD on COCO minival split. ResNet-101 is used as the backbone. As shown in Figure 8, our proposed FFAD can perform well in various natural scenes, being urban, wild, land, or air. A wide range of objects can be detected by FFAD, including crowded, incomplete, extremely small, and very large objects.

### 4.8. Generalization on Global Wheat Head Detection

In addition to evaluation on the COCO dataset, we further corroborate the proposed method on the Global Wheat Head Detection (GWHD) dataset [55]. The public dataset brings about a challenging task for detecting wheat head from several countries around the world at different growth stages with a wide range of genotypes. To further verify and delve the effectiveness of our proposed algorithm, we run FFAD and several other detectors including Faster RCNN [19], Mask RCNN [21], RetinaNet [25], FCOS [26], EfficientDet [5], and YOLOv5 [56] on this dataset. We separate out a fifth of the training set as a validation set and then evaluate the results on that. We set the input size to 1024 × 1024 and the batch size to 4 to train these models for 10 epochs. As shown in Table 7, even in the face of such dense and overlapping scenes, FFAD can still give satisfactory improvements.

## 5. Conclusion and Future Work

In this paper, we point out that there are several bottlenecks existing in current feature pyramid networks, which considerably limit the performance of detectors. Motivated by that, we look into these issues and propose a novel feature pyramid network with feature fusion and disentanglement (FFAD) to alleviate these problems. In particular, FFAD first splits the conventional bidirectional feature pyramid into two independent subnetworks and adds an additional flow of information to each of them to strengthen the communication between feature maps and finally fuses the output of the two subnetworks. Furthermore, we propose the task-adaptive convolution to mitigate the inherent task conflict in feature pyramid. By predicting two groups of different offsets and modulations in task-adaptive convolution, FFAD generates the specific feature representation for classification and localization, respectively. Being compatible with most single-stage object detectors, our FFAD can easily enhance the detection performance by about 1∼2.6 AP. Our future work will aim to simplify feature fusion module without losing mAP and further enlarge the performance margin between the disentangled and the shared features in pyramidal model.

## Figures and Tables

**Table 1:** Comparison of our method with other fashion feature fusion modules including FPN, bidirectional FPN, and encoder-decoder FPN on FCOS with ResNet-50 backbone. Results evaluated on MS-COCO minival are reported.

Method	AP	AP_S_	AP_M_	AP_L_
FPN	37.1	21.3	41.0	47.8
Encoder-decoder FPN	37.3	21.6	41.4	48.1
Bidirectional FPN	37.6	21.8	41.3	48.4
3-flow structure	37.8	22.0	41.7	49.8
5-flow structure	38.3	22.8	42.3	49.7
MF^3^M	**39.2**	**23.3**	**42.8**	**51.2**

**Table 2:** Ablation studies on our proposed task-adaptive convolution.

Method	AP	AP_50_	AP_75_
FCOS	37.1	55.9	39.8
FCOS + MF^3^M	39.2	57.8	42.2
FCOS + MF^3^M + TaConv	39.7	58.2	43.5

All of the experiments are trained on FCOS with ResNet-50 backbone.

**Table 3:** Comparison of detection AP results of MF^3^M with different DCN's positions.

DCN's position	AP	AP_S_	AP_M_	AP_L_
P1	39.0	23.1	42.3	50.5
P2	39.2	23.3	42.8	51.2
P3	38.7	21.6	41.5	49.9

All experiments were trained on FCOS with ResNet-50 backbone. Results evaluated on MS-COCO minival are reported.

**Table 4:** Comparison of detection AP results of different architectures.

Method	Testing time (ms)	AP	AP_50_	AP_75_	AP_S_	AP_M_	AP_L_
RetinaNet	56	35.7	55.0	38.5	18.9	38.9	46.3
RetinaNet + FFAD	58	**37.5**	**57.1**	**40.4**	**22.7**	**41.2**	**49.3**
FCOS	45	37.1	55.9	39.8	21.3	41.0	47.8
FCOS + FFAD	48	**39.7**	**58.2**	**43.5**	**24.7**	**43.5**	**52.2**
ATSS	44	39.3	57.5	42.8	24.3	43.3	51.3
ATSS + FFAD	49	**41.4**	**59.1**	**45.0**	**25.1**	**45.5**	**53.8**

All models were trained using ResNet-50 backbone and the same training strategies. Results are evaluated on COCO minival set.

**Table 5:** Comparison of FFAD with other state-of-the-art feature pyramid networks. Results are evaluated on COCO minival set.

Pyramidal models	FLOPS (G)	AP	AP_50_	AP_75_
FPN	200.04	37.1	55.9	39.8
PANet	216.58	37.8	57.1	41.2
Libra	275.62	38.0	58.3	40.8
HRNet	258.03	38.1	58.2	41.3
NAS-FPN	249.09	38.9	57.6	42.6
FFAD	230.79	39.7	58.2	43.5

**Table 6:** Comparison of the test results of FFAD with other state-of-the-art object detectors. Results are evaluated on COCO test-dev.  ∼ indicates multiscale testing is used.

Method	Backbone	AP	AP_50_	AP_75_	AP_S_	AP_M_	AP_L_
*Two-stage detectors*							
Faster RCNN w/FPN [19]	ResNet-101	36.2	59.1	39.0	18.2	39.0	48.2
Deformable R–FCN [40]	Inc-Res-v2	37.5	58.0	40.8	19.4	40.1	52.5
Mask-RCNN [21]	ResNext-101	39.8	62.3	43.4	22.1	43.2	51.2
Soft-NMS [41]	ResNet-101	40.8	62.4	44.9	23.0	43.4	53.2
SOD-MTGAN [42]	ResNet-101	41.4	63.2	45.4	24.7	44.2	52.6
Cascade-RCNN [43]	ResNet-101	42.8	62.1	46.3	23.7	45.5	55.2
TridentDet [44]	ResNet-101	42.7	63.6	46.5	23.9	46.6	56.6
TSD [11]	ResNet-101	43.2	64.0	46.9	24.0	46.3	55.8
SNIP^∼^ [1]	DCN + ResNet-101	44.4	66.2	49.2	27.3	46.4	56.9
SNIPER^∼^ [29]	DCN + ResNet-101	46.1	67.6	51.5	28.0	51.2	60.5

*One-stage detectors*							
DSSD513 [45]	ResNet-101	33.2	53.3	35.2	13.0	35.4	51.1
RefineDet512 [46]	ResNet-101	36.4	57.5	39.5	13.6	39.9	51.4
RetinaNet800 [25]	ResNet-101	39.1	59.1	42.3	21.8	42.7	50.2
PPDet [47]	ResNet-101	40.7	60.2	44.5	24.5	44.4	49.7
AutoFPN [48]	ResNet-101	42.5	-	-	-	-	-
FreeAnchor [49]	ResNet-101	43.0	62.2	46.4	24.7	46.0	54.0
M2Det ^∼^ [7]	ResNet-101	43.9	64.4	48.0	29.6	49.6	54.3
FoveaBox [50]	ResNext-101	42.1	61.9	45.2	24.9	46.8	55.6
FCOS [26]	ResNext-101	44.7	64.1	48.4	27.6	47.5	55.6
CornerNet [51]	Hourglass-104	40.6	56.4	43.2	19.1	42.8	54.3
ExtremeNet [52]	Hourglass-104	40.1	55.3	43.2	20.3	43.2	53.1
CenterNet [53]	Hourglass-104	44.9	62.4	48.1	25.6	47.4	57.4
CenterNet ^∼^ [53]	Hourglass-104	47.0	64.5	50.7	28.9	49.9	58.9
RepPoints [54]	DCN + ResNet-101	45.0	66.1	49.0	26.6	48.6	57.5

*Ours*							
FFAD	ResNet-101	**44.1**	62.2	47.9	27.4	47.6	56.7
FFAD	DCN + ResNet-101	**46.5**	64.9	51.2	29.3	51.3	60.8
FFAD	DCN + ResNext-101	**47.4**	66.9	52.0	31.1	51.5	61.9
FFAD ^∼^	DCN + ResNext-101	**49.5**	**68.9**	**53.9**	**35.8**	**53.6**	**63.3**

**Table 7:** Comparison of the test results of FFAD with other state-of-the-art object detectors.

Method	Backbone	mAP@0.5
Faster RCNN	ResNet-50	80.8
Mask RCNN	ResNet-50	83.6
RetinaNet	ResNet-50	87.5
FCOS	ResNet-50	88.6
EfficientDet (D3)	ResNet-50	88.9
YOLOv5	CSPDarknet	89.3
FFAD	ResNet-50	90.9

Results are evaluated on GWHD.

## Data Availability

The data were obtained from the following public dataset MS COCO: http://images.cocodataset.org/zips/train2017.zip (for training), http://images.cocodataset.org/zips/val2017.zip (for validation), and http://images.cocodataset.org/zips/test2017.zip (for testing).

## References

[1] Singh B., Davis L. S. An analysis of scale invariance in object detection snip.

[2] Liu W. (2016). Ssd: single shot multibox detector. *European Conference on Computer Vision*.

[3] Lin T.-Yi Feature pyramid networks for object detection.

[4] Wang K. Panet: few-shot image semantic segmentation with prototype alignment.

[5] Tan M., Pang R., Quoc V. Efficientdet: scalable and efficient object detection.

[6] Pang J. Libra r-cnn: towards balanced learning for object detection.

[7] Zhao Q., Sheng T., Wang Y. (2019). M2det: a single-shot object detector based on multi-level feature pyramid network. *Proceedings of the AAAI Conference on Artificial Intelligence*.

[8] Wang X. Scale-equalizing pyramid convolution for object detection.

[9] Jiang B. Acquisition of localization confidence for accurate object detection.

[10] Wu Y. Rethinking classification and localization for object detection.

[11] Song G., Liu Yu, Wang X. Revisiting the sibling head in object detector.

[12] Hu J., Shen Li, Sun G. Squeeze-and-excitation networks.

[13] Woo S., Park J., Lee J.-Y., Kweon I. S. (2018). CBAM: convolutional block attention module. *Computer Vision-ECCV 2018*.

[14] Zhu X. An empirical study of spatial attention mechanisms in deep networks.

[15] Guo H. (2020). Scene-driven multitask parallel attention network for building extraction in high-resolution remote sensing images. *IEEE Transactions on Geoscience and Remote Sensing*.

[16] Peng J. W., Jiang F., Chen H., Zhou Y., Du Q. (2020). A general loss-based nonnegative matrix factorization for hyperspectral unmixing. *IEEE Geoscience and Remote Sensing Letters*.

[17] Sun R. Rich feature hierarchies for accurate object detection and semantic segmentation.

[18] Girshick R. Fast R-CNN.

[19] Ren S. (2015). Faster R-CNN: towards real-time object detection with region proposal networks. *Advances in Neural Information Processing Systems*.

[20] Dai J. (2016). R-FCN: object detection via region-based fully convolutional networks. *Advances in Neural Information Processing Systems*.

[21] He K. Mask R-CNN.

[22] Redmon J. You only look once: unified, real-time object detection.

[23] Redmon J., Ali F. YOLO9000: better, faster, stronger.

[24] Redmon J., Ali F. (2018). Yolov3: an incremental improvement. http://arxiv.org/abs/1804.02767.

[25] Lin T.-Y. Focal loss for dense object detection.

[26] Tian Z. FCOS: fully convolutional one-stage object detection.

[27] Zhu C., He Y., Savvides M. Feature selective anchor-free module for single-shot object detection.

[28] Zhang S. Bridging the gap between anchor-based and anchor-free detection via adaptive training sample selection.

[29] Singh B., Najibi M., Larry S., Davis Sniper: efficient multi-scale training.

[30] Liu S., Huang D. Receptive field block net for accurate and fast object detection.

[31] Newell A., Yang K., Jia D. (2016). Stacked hourglass networks for human pose estimation. *European Conference on Computer Vision*.

[32] Sun Ke Deep high-resolution representation learning for human pose estimation.

[33] Zhang H., Wang J. Towards adversarially robust object detection.

[34] Zhu X. Deformable convnets v2: more deformable, better results.

[35] Lin T.-Yi (2014). Microsoft coco: common objects in context. *European Conference on Computer Vision*.

[36] Lu X. Grid R-CNN.

[37] Russakovsky O., Deng J., Su H. (2015). Imagenet large scale visual recognition challenge. *International Journal of Computer Vision*.

[38] He K. Deep residual learning for image recognition.

[39] Ghiasi G., Lin T.-Yi, Quoc V., Le Nas-Fpn: learning scalable feature pyramid architecture for object detection.

[40] Dai J. Deformable convolutional networks.

[41] Bodla N. Soft-NMS--improving object detection with one line of code.

[42] Bai Y. Sod-MTGAN: small object detection via multi-task generative adversarial network.

[43] Cai Z., Vasconcelos N. Cascade R-CNN: delving into high quality object detection.

[44] Li Y. Scale-aware trident networks for object detection.

[45] Fu C.-Y. (2017). DSSD: deconvolutional single shot detector. http://arxiv.org/abs/1701.06659.

[46] Zhang S. Single-shot refinement neural network for object detection.

[47] Samet N., Hicsonmez S., Akbas E. (2020). Reducing label noise in anchor-free object detection. http://arxiv.org/abs/2008.01167.

[48] Xu H. Auto-FPN: automatic network architecture adaptation for object detection beyond classification.

[49] Zhang X. (2019). Freeanchor: learning to match anchors for visual object detection. *Advances in Neural Information Processing Systems*.

[50] Kong T., Sun F., Liu H., Jiang Y., Li L., Shi J. (2020). FoveaBox: beyound anchor-based object detection. *IEEE Transactions on Image Processing*.

[51] Law H., Jia D. Cornernet: detecting objects as paired keypoints.

[52] Zhou X., Zhuo J., Krahenbuhl P. Bottom-up object detection by grouping extreme and center points.

[53] Duan K. Centernet: keypoint triplets for object detection.

[54] Yang Z. Reppoints: point set representation for object detection.

[55] David E. (2020). Global Wheat Head Detection (GWHD) dataset: a large and diverse dataset of high resolution RGB labelled images to develop and benchmark wheat head detection methods. http://arxiv.org/abs/2005.02162.

[56] Jocher G., Stoken A., Borovec J. (2020). *ultralytics/yolov5: v3.1-Bug Fixes and Performance Improvements (Version v3.1)*.

